# Clinicopathological Features and Outcomes of Endometrial Cancer: A single institution experience

**DOI:** 10.18295/squmj.3.2024.014

**Published:** 2024-05-27

**Authors:** Ikram A. Burney, Shahd Al Ghafri, Jawahar Al Noumani, Anisa Al Jabri, Anjum O. Hasan, Sarya Bella, Hasan Al-Sayegh, Radhiya Al Ajmi, Moza Al Kalbani

**Affiliations:** 1 Women Health Program, Sultan Qaboos Comprehensive Cancer Care and Research Center, Muscat, Oman; 2 Department of Surgery, The Royal Hospital, Muscat, Oman; 3 Department of Medicine, The Royal Hospital, Muscat, Oman; 4 Department of Radiation Oncology, The Royal Hospital, Muscat, Oman; 5 Department of Research Laboratories, Sultan Qaboos Comprehensive Cancer Care and Research Center, Muscat, Oman; 6 Pathology, Sultan Qaboos University Hospital, Sultan Qaboos University, Muscat, Oman

**Keywords:** Endometrial Cancer, Obesity, Cancer, Survival, Oman

## Abstract

**Objectives:**

This study aimed to report the demographic features, clinical presentation, pathological types and long-term outcomes of patients diagnosed with endometrial cancer (EC) in Oman. EC is the sixth most common cancer in women worldwide and the fifth most common cancer in women in Oman. Survival outcomes of EC have not been reported previously from Oman.

**Methods:**

This retrospective study was carried out on consecutive patients treated at the Sultan Qaboos University Hospital, Muscat, Oman, between 2008 and 2020. Survival was estimated using the Kaplan and Meier method.

**Results:**

A total of 50 patients with EC were included. The median age was 61 years (range: 31–86 years), and 72% of the patients had type I histology. Most patients were diagnosed with stage IA and IB EC (49% and 20%, respectively), and the majority had grade 1 or 2 tumours (40% and 34%, respectively). Overall, the 5-year survival and 10-year survival rates were estimated to be 70% and 56%, respectively. Weight (>75 kg) and body mass index (>30 kg/m^2^) were significantly associated with better survival. Tumour histology (type I versus type II or carcinosarcoma), grade (1 versus 2 versus 3) and stage (IA or IB versus II–IV) were associated with better overall survival (*P* = 0.007, *P* <0.0001 and *P* <0.0003, respectively). Patients diagnosed with EC with co-morbidities, other than obesity, had inferior survival compared to those without co-morbidities.

**Conclusion:**

Median age at presentation, histological sub-type, clinical stage and outcomes are comparable to the published literature. Almost two-thirds of the patients were obese. These data could be used as a benchmark for outcomes of EC in the region.

**Advances in Knowledge**

- *In Oman, the outcomes of patients diagnosed with endometrial cancer are comparable to the published literature from the region and internationally.*
- *Almost two-thirds of the patients are obese at the time of diagnosis.*
- *Patients who are overweight and obese have a better prognosis, as the vast majority have the endometroid type of endometrial cancer.*

**Applications to Patient Care**

- *Approximately 50% of patients are diagnosed with stage I disease at presentation and surgical treatment suffices.*
- *All other patients require adjuvant radiotherapy, chemotherapy, both or palliative treatment.*
- *The data presented in this study could be used as a benchmark for the outcomes of endometrial cancer in the region.*

Endometrial cancer (EC) is the sixth most common cancer in women worldwide, with an incidence of 10.1 per 100,000 and a mortality rate of 2.4 per 100,000 patients.^1^ Incidence rates vary in different parts of the world, with EC being the most common gynaecological cancer in the West.^2^^,^^3^ In the last 2 decades, an increase in the incidence of EC has been reported, possibly related to the rising prevalence of obesity. Obesity may increase the risk of EC by 2.6-fold, and with severe obesity, the risk increases by 4.6-fold.^4^ There are several other risk factors, which predispose women to EC, and these are classifiable into two groups. Modifiable risk factors include pelvic radiation therapy, duration of menstruation, late menopause, early menstruation, diabetes, fatty diet, polycystic ovarian disease, supplements, tamoxifen, pregnancy and endometrial hyperplasia. Non-modifiable risk factors include age and family history. A family history of EC increases the risk by 2- to 3-fold.^5^

EC can be classified into two major sub-types. Type I or endometroid adenocarcinoma accounts for approximately 80% of all EC; type II carcinoma accounts for 15–20%, including serous carcinoma, clear cell carcinoma and carcinosarcoma.^6^ Type I EC are usually oestrogen-receptor positive, present with localised disease and have a favourable prognosis, whereas, type II EC usually do not express oestrogen-receptor, present with advanced stage disease and have a poor prognosis.^7^ The 5-year survival rate among patients with metastatic disease has been reported to be around 17%.^8^ More recently, EC has been classified according to the molecular profile. Subtypes include POLE-ultra mutated, which has the best prognosis, mismatch repair-deficient and no specific molecular profile EC, both of which have an intermediate prognosis, and p53-abnormal, which has the worst prognosis.^9^

EC is the fifth most common cancer in women in Oman after breast, thyroid, colorectal and stomach cancers.^10^ There is a geographical variation in the incidence and presentation of EC worldwide. For example, mutation frequency profiles for different ethnicities and tumour grades in EC patients revealed a higher mutation frequency for *PIK3CA* and *ARID1A* in White and Asian patients; *TP53* and *FAT1* in Black/African Americans; and *CTNNB1* and *RYR2* in Native Hawaiians or Asians.^11^ Important variations in incidence and mortality rates of EC have also been reported over the last 3 decades.^12^ Hence, it is important to report the presenting features and outcomes of EC patients in Oman and the region. The study aimed to report the demographic features, clinical presentation, pathological types and long-term outcomes of patients with EC in Oman.

## Methods

This retrospective study included consecutive patients diagnosed with EC and treated at the Sultan Qaboos University Hospital (SQUH), Muscat, Oman. SQUH was one of the two referral centres for cancer treatment in Oman. Patients diagnosed with uterine sarcoma, lymphoma or metastatic disease were excluded. Electronic patient records (EPR) of patients diagnosed with EC between 2008 and 2020 were reviewed, and demographic features including age and comorbidities were extracted. Body mass index (BMI) was calculated using the height and weight of the patient at the time of diagnosis. A patient was defined to have diabetes, hypertension, ischaemic heart disease (IHD) or hyperlipidaemia if the illness had been noted in the EPR or the patient was receiving treatment for these conditions at the time of diagnosis. Information on histological subtypes and tumour grade was extracted from the archived notes and verified by a single pathologist. Overall survival (OS) outcomes were measured from the date of diagnosis to the date of death for deceased patients or the date of last follow-up (on or before December 31, 2021) for censored patients.

Median and range were reported for the continuous variables; frequency and proportions were reported for the categorical variables. The 5-year OS estimates were calculated using the Kaplan-Meier method.^13^ Comparisons of study groups were performed using the log-rank test. A *P* value of ≤0.05 was considered statistically significant. Analysis was performed using the SAS software, Version 9.4 (SAS Institute Inc., Cary, North Carolina, USA).

The study was approved by the Institutional Medical Research Ethics Committee.

## Results

A total of 50 patients were diagnosed with EC and all were included in the analysis. The median age was 61 years (range = 31–86 years). Median weight was 76 kg (range = 34–126 kg). The mean BMI was 34 kg/m^2^, and 62% of patients were obese (BMI ≥30 kg/m^2^). There were 36 (72%) patients who were diagnosed with type I tumours, and 4 (8%) patients with carcinosarcoma. Most patients presented with stage IA and IB disease (49% and 20%, respectively), and most patients had grade 1 and 2 tumours (40% and 34%, respectively) [Table 1]. A total of 13 patients died during the follow-up time with a median time from diagnosis to death being 2 years (range = 4 months–5.8 years). Among the patients, 37 survived to the last follow-up with a median follow-up time of 3.4 years. OS was 70 ± 8% at 5 years and 56 ± 11% at 10 years from diagnosis.

**Figure 1:**
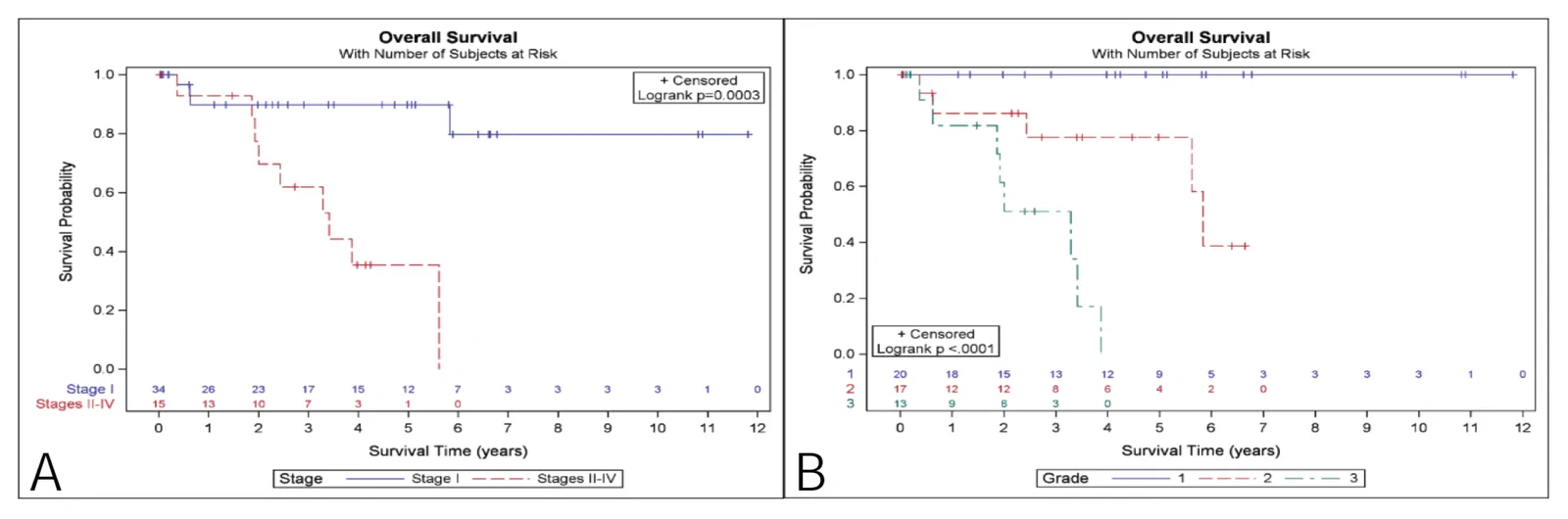
Overall survival of patients with endometrial cancer by **(A)** tumour stage and **(B)** tumour grade.

**Figure 2:**
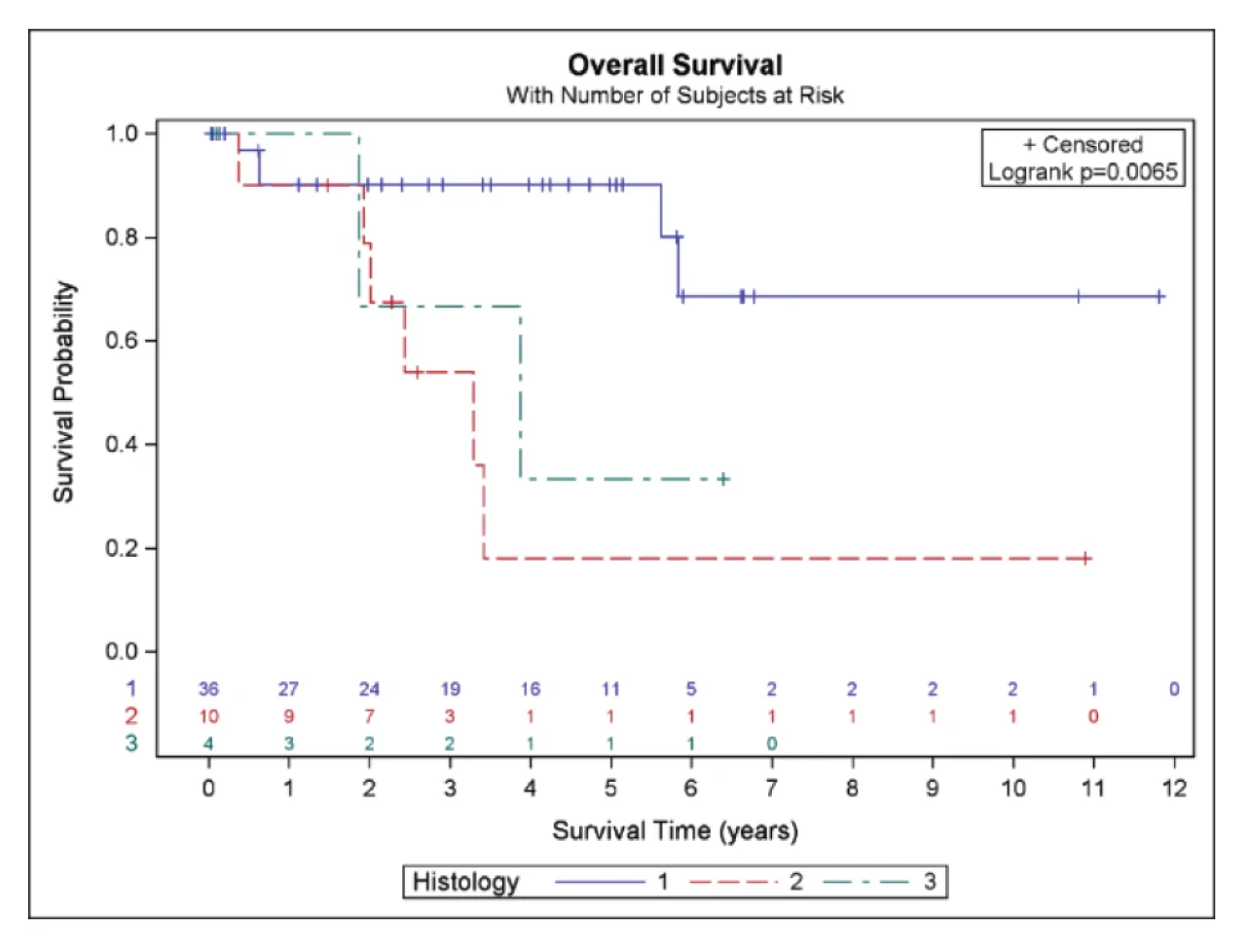
Overall survival of patients with endometrial cancer by histological type.

**Figure 3:**
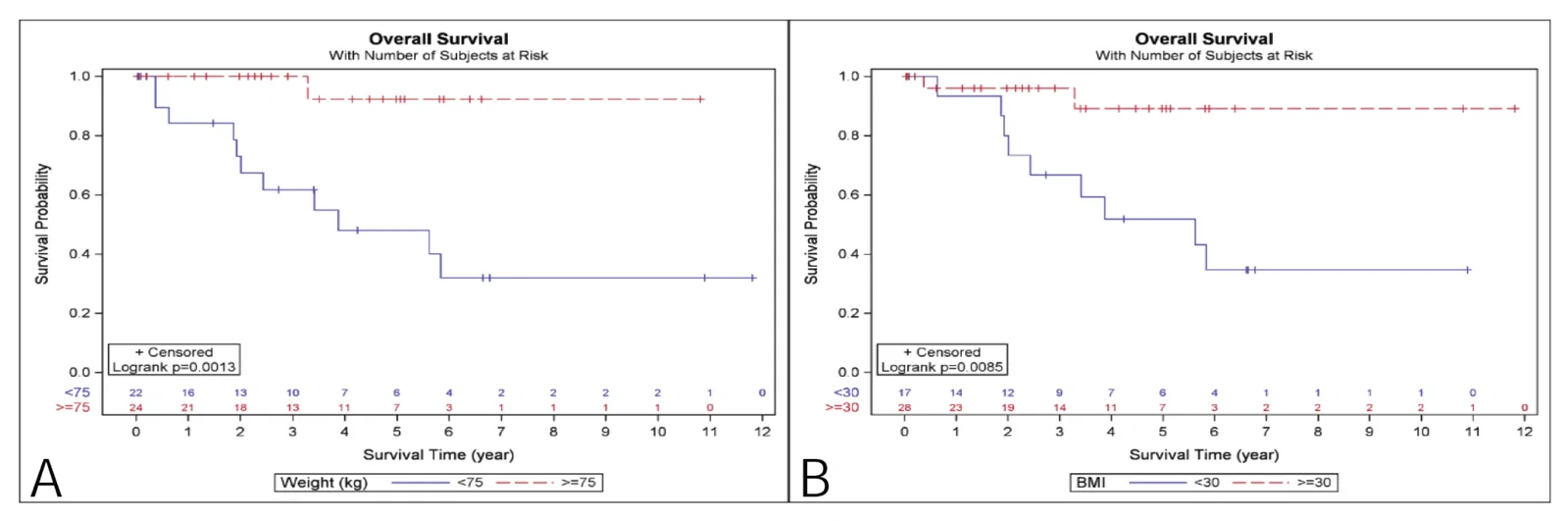
Overall survival of patients with endometrial cancer by **(A)** weight and **(B)** body mass index.

**Figure 4:**
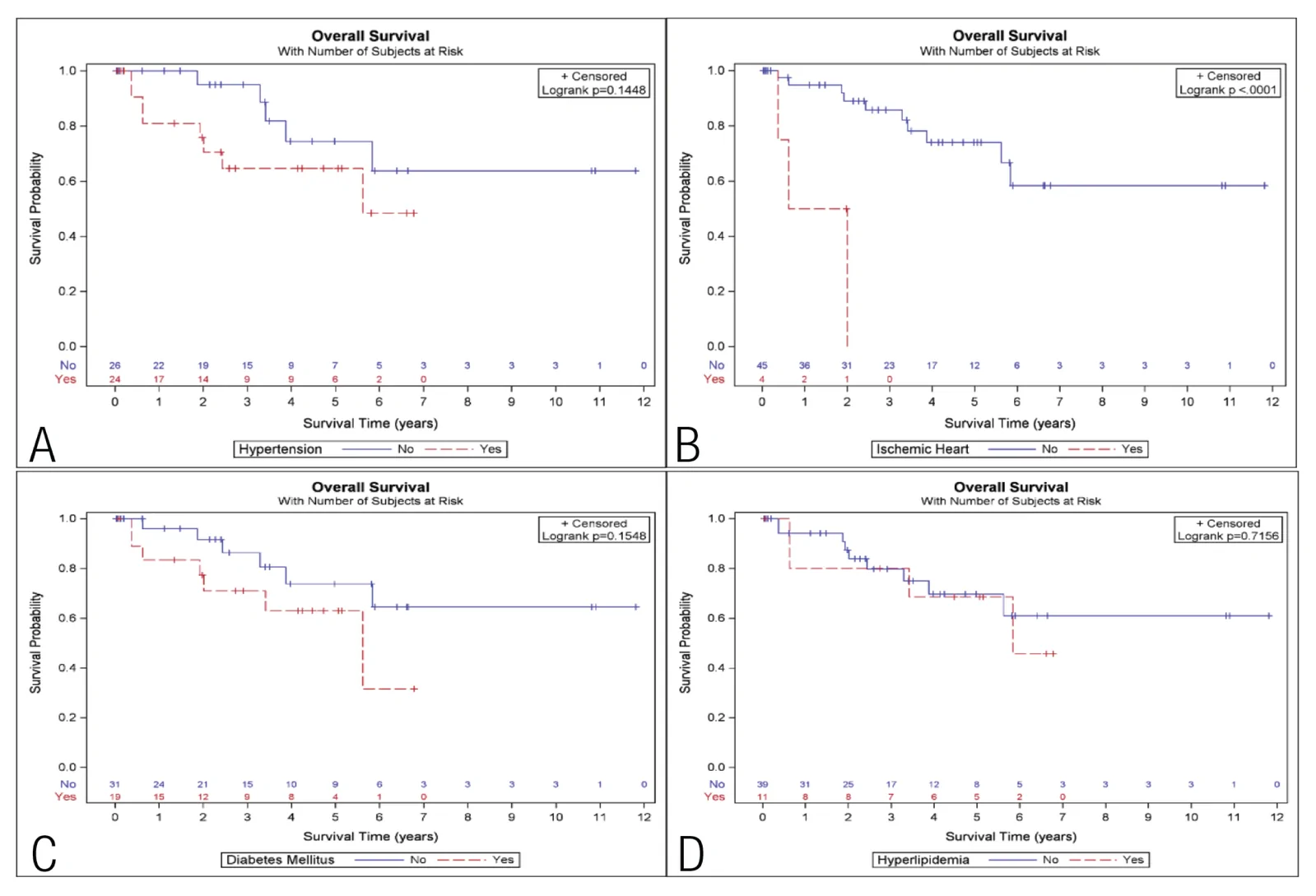
Overall survival of patients with endometrial cancer by **(A)** hypertension, **(B)** ischaemic heart disease, **(C)** diabetes mellitus and **(D)** hyperlipidaemia.

Table 2 and Figures 1–4 show OS outcomes. Patients who weighed more than 75 kg at diagnosis had a 92 ± 7% OS rate at 4 years compared to 48 ± 12% for patients who weighed less than 75 kg (*P* = 0.001); 28 patients were obese (BMI ≥30 kg/m^2^) and had a better 5-year survival compared to those with BMI less than 30 kg/m^2^ (89% versus 52%; *P* = 0.009). The OS outcomes were also significantly associated with the tumour histology (*P* = 0.007), grade (*P* <0.0001) and stage I versus II–IV (*P* <0.0003) [Table 2]. History of IHD was associated with a statistically significant worse survival. Patients with IHD (n = 4) had an OS of 50 ± 25% at 2 years and 0% at 5 years from diagnosis compared to 89 ± 5% and 74 ± 8% for patients without IHD (n = 45) [Figures 1–4].

## Discussion

This is the first study reporting the demographic, pathological and clinical features at presentation and outcomes after treatment of EC from Oman. EC is the most common gynaecological cancer in the Gulf Cooperation Council (GCC) region and globally. Data regarding EC are available from tumour registries from several member states of the GCC.^10^ However, these data are limited, because they report only the incidence, location of the patients, age and histological subtypes.^10^ There are no studies on the presenting features, presence of comorbidities, clinical stage and long-term survival of patients from the GCC. However, a few studies from Turkey and Saudi Arabia have been published.^14^^,^^15^ The median age of patients at diagnosis with EC in the current study was 61 years, which is comparable with the registry data from Saudi Arabia (60 years) and also with reports from the Western literature (50–70 years).^16^^,^^17^

Almost two-thirds of the patients in the current study were obese. This result conforms with the studies published in the USA, which reported that 72% of the patients were either overweight or obese.^18^ Obesity is an important modifiable risk factor in EC and cancers of the gall bladder, oesophagus, kidney and post-menopausal breast.^19^^,^^20^ In this cohort, obese patients had significantly better survival than patients with a BMI of less than 30 kg/m^2^. The relationship between obesity and mortality in patients with EC has been a subject of debate. On one hand, every 5 kg/m^2^ increase in BMI has been shown to confer an increased risk of EC; however, obesity-driven ECs are usually type I, low grade and are diagnosed at an early stage.^18^ On the other hand, obesity predisposes women to a range of comorbidities including diabetes mellitus, hypertension and IHD. Women with a BMI of more than 35 kg/m^2^ have been reported to have an almost 5-fold higher risk of cardiovascular-related mortality 10 years after diagnosis of EC.^21^ Women with a BMI ≥40 kg/m^2^ have significantly higher odds of all-cause mortality. There are no consistent reports of an association between diabetes mellitus and EC-related mortality.^22^^,^^23^ Furthermore, obesity may affect the safe and effective delivery of treatment. For example, obese patients are less likely to be offered a hysterectomy and may receive sub-optimal doses of radiotherapy and chemotherapy.^24^ In this cohort, 48%, 38%, 22% and 8% of patients had hypertension, diabetes mellitus, dyslipidaemia and IHD, respectively. However, only patients with IHD had significantly inferior survival compared to those who did not have IHD.

All patients received treatment based on the National Comprehensive Cancer Network guidelines.^25^ Based on clinical stage and pathological and molecular factors, EC can be classified into low-risk, intermediate-risk, high-intermediate risk, high-risk and advanced metastatic disease.^26^ Low-risk EC does not need to be treated with adjuvant treatment after surgery. The role of adjuvant chemotherapy is controversial in EC.^27^^,^^28^ Even though early-stage EC has a better prognosis, 5–30% of cases experience distant metastasis. More than 70% of type II EC develop distant metastasis. Adjuvant chemotherapy does not improve 5-year OS for patients with high-risk EC, but it increases failure-free survival. Hence, the treatment should be individualised after shared decision-making.^29^

The current study has several limitations. First, the study covered a long period of 12 years but the standards of care did not change significantly over the study period; this factor is unlikely to change the results of the study in terms of survival outcomes. For example, molecular classification was first reported in 2013 but was not used until 2020 in routine clinical practice, thus not having an influence on treatment decisions.^9^ Immune checkpoint inhibitors were approved for use in recurrent EC only in 2020.^30^ Second, this study was retrospective and is subject to biases inherent in retrospective data collection. Third, the sample size was relatively small (N = 50). However, results support and conform with previously published studies, both regionally and internationally. Finally, this study reports the experience of a single centre. However, patients diagnosed with cancer in Oman receive the initial treatment in one of the two hospitals, and both are located in the capital (Muscat). The patients are referred either to the Ministry of Health hospitals or SQUH. Since patients are received from all over the country in SQUH, it may be plausible to assume that the pattern of presentation and outcomes reflect the situation in the country.

## Conclusion

Median age at presentation, histological sub-type, clinical stage and survival outcomes among patients with EC in Oman are comparable to the published literature in a global context. Histological subtype, degree of differentiation and clinical stage were associated with survival. Almost two-thirds of patients were obese and had better OS because the disease had good prognostic factors. These data could be used as a benchmark for outcomes of EC in the region.

## Figures and Tables

**Table 1:** Characteristics of patients with endometrial cancer at Sultan Qaboos University Hospital, Muscat, Oman (N = 50)

Characteristic	n (%)
**Median age years (range)**	61 (31–86)
**Median weight in kg (range)**	76 (34–126)
**Median height in cm (range)**	152 (131–165)
**Mean BMI in kg/m** **^2^** ** (range)**	34 (15–67)
**BMI category in kg/m** **^2^**	
<18 (underweight)	1 (2)
18–<24 (normal weight)	6 (13)
25–<30 (overweight)	10 (22)
≥30 (obese)	28 (62)
**Histology**	
Type I	36 (72)
Type II	10 (20)
**Carcinosarcoma**	4 (8)
**Grade**	
1	20 (40)
2	17 (34)
3	13 (26)
**Stage**	
IA	24 (49)
IB	10 (20)
2	4 (8)
3	8 (16)
4	4 (8)
**Hypertension**	24 (48)
**IHD**	4 (8)
**Hyperlipidaemia**	11 (22)
**Diabetes mellitus**	19 (38)

BMI = body mass index; IHD = ischaemia heart disease.

**Table 2:** 5-year overall survival of patients with endometrial cancer.

Characteristic	n	5-year in % OS ± SE	Log-rank test *P* value^*^
**Age in years**			0.3
<60	21	73 ± 12	
≥60	29	68 ± 10	
**Weight in kg**			0.001
<75	22	48 ± 12	
≥75	24	92 ± 7	
**Height in cm**			0.4
<150	15	72 ± 14	
≥150	30	72 ± 10	
**BMI in kg/m** **^2^**			0.009
<30	17	52 ± 13	
≥30	28	89 ± 8	
**Histology**			0.007
Type I	36	90 ± 5	
Type II	10	18 ± 16	
Type III	4	33 ± 27	
**Grade**			<0.0001
1	20	100 ± 0	
2	17	78 ± 12	
**Carcinosarcoma**	13	0	
**Stage**			<0.0003
IA/IB	34	90 ± 5	
II–IV	16	35 ± 13	
**Hypertension**			0.14
Yes	24	65 ± 11	
No	26	74 ± 11	
**IHD**			<0.0001
Yes	4	0 (NA)	
No	45	74 ± 8	
**Hyperlipidaemia**			0.7
Yes	11	69 ± 15	
No	39	70 ± 9	
**Diabetes mellitus**			0.2
Yes	19	63 ± 12	
No	31	74 ± 10	

OS = overall survival; SE = standard error; BMI = body mass index; IHD = ischaemic heart disease; NA = not available (value cannot be estimated).
